# Chronological and Carbohydrate-Dependent Transformation of Fatty Acids in the Larvae of Black Soldier Fly Following Food Waste Treatment

**DOI:** 10.3390/molecules28041903

**Published:** 2023-02-16

**Authors:** Yanxia Liu, Junliang Liu, Jinwen He, Hongxu Lu, Shibo Sun, Fengyun Ji, Xiaoying Dong, Yongming Bao, Jianqiang Xu, Gaohong He, Weiping Xu

**Affiliations:** 1 School of Ocean Science and Technology (OST) & Panjin Institute of Industrial Technology (PIIT), Dalian University of Technology, Panjin 124221, China; 2 School of Life and Pharmaceutical Sciences (LPS), Dalian University of Technology, Panjin 124221, China; 3 State Key Laboratory of Fine Chemicals, School of Chemical Engineering, Dalian University of Technology, Dalian 116024, China

**Keywords:** *Hermetia illucens*, food waste, fatty acids, carbohydrates, bioconversion, chronological changes

## Abstract

Although black soldier fly larvae (BSFL) can convert food waste into insectile fatty acids (FAs), the chronological and diet-dependent transformation of larval FAs has yet to be determined. This study focused on the dynamics of larval FA profiles following food waste treatment and characterized factors that may drive FA composition and bioaccumulation. Larval FA matters peaked on Day 11 as 7.7 ± 0.7% of food waste dry matter, maintained stably from Day 11–19, and decreased slightly from Day 19–21. The BSFL primarily utilized waste carbohydrates for FA bioconversion (Day 0–11) and shifted to waste FAs (Day 7–17) when the carbohydrates were close to depletion. The optimal time window for larvae harvest was Days 17–19, which fulfilled both targets of waste oil removal and larval FA transformation. Larval FAs were dominated by C12:0, followed by C18:2, C18:1, and C16:0. The waste-reducing carbohydrate primarily accounted for larval FA bioaccumulation (r = −0.947, *p* < 0.001). The increase in diet carbohydrate ratio resulted in the elevation of larval C12:0 yield, which indicated that larval C12:0-FA was primarily biosynthesized from carbohydrates and further transformed from ≥C16 FAs. This study elucidates the bioaccumulation process of larval FAs for food waste treatment and highlights the importance of waste carbohydrates for both the composition and transformation of larval FAs.

## 1. Introduction

The United Nations Environment Programme estimates that the annual global production of food waste is currently about 931 million tons [1]. In 2021, the domestic waste production in China reached 235 million tons, of which approximately 117–141 million tons were food waste [2]. The conventional methods of food waste treatment comprise landfill, incineration, composting, and anaerobic biogas. The insectile treatment is a novel method for food waste valorization, and the utilization of black soldier fly (BSF, *Hermetia illucens*) has been studied and applied in recent years [3,4]. Larvae of black soldier fly (BSFL) can feed on food waste and convert solid waste into animal feed and organic fertilizer [5,6,7,8]. This process enables the efficient recycling of organic waste, which fulfills the United Nations’ concept of Sustainable Development Goal 12: Sustainable Consumption and Production [9].

The BSFL biomass is composed of proteins, fatty acids (FAs), chitin, and other biological molecules. The BSFL FAs can be used in the preparation of feed oils, biodiesel, and soap detergents [10,11,12]. Thus, the bioconversion of food waste into insectile FAs has an important economic value and waste recycling significance. There are several studies focusing on the dynamics of larval FA profiles based on animal feed substrates; however, there is rear research that examined chronological changes of larval FA profiles following waste treatment. Liu et al. [13] and Zhu et al. [14] reported temporal variations of larval FA profiles with the substrate of chicken feed. Li et al. [15] investigated temporal changes in larval FA yield based on standard feed supplied with glucose or xylose. However, the substrate component substantially affected larval FA composition and transformation performance [10,11,12]. Due to the distinct composition between food waste and animal feed, the chronological profiles of larval FAs following food waste treatment, as well as the bioconversion process, should be deeply investigated in order to facilitate the industrial application of BSFL technology for food waste valorization.

The BSFL-derived FAs have been successfully used in chicken and fish feeds [16,17,18,19,20]. The larval FA content accounts for approximately 15–49% of the body dry mass [20], which is dominated by lauric acid (C12:0) and followed by myristic acid (C14:0), palmitic acid (C16:0), palmitoleic acid (C16:1), stearic acid (18:0), oleic acid (C18:1), linoleic acid (C18:2) and linolenic acid(C18:3) [14,21]. Among the FA species, the lauric acid (C12:0) has shown antimicrobial activities [22,23,24,25,26], while the mono- and polyunsaturated fatty acids (MUFAs, PUFAs) are acknowledged with better nutritional effects than saturated fatty acids (SFAs). Based on the chicken feed substrate, Liu et al. [13] and Zhu et al. [14] found that the larval FAs rapidly developed along with the larval and prepupal stage and greatly decreased in the pupal stage; the C12:0 FA was the predominant FA species with its proportion increasing from 16.4% to 62.5%, while the C14:0, C16:0, C18:1, and C18:2 FA were minor species with their individual proportions declining from 31.4% to 4.6%. The application of larval FAs in the feed oil sector requires the proportion of UFAs to be elevated as much as possible. The food waste substrate may fulfill these needs; however, more research should be carried out to reveal the UFA proportions in BSFL following food waste treatment [27,28].

Food waste contains different macronutrients such as carbohydrates, lipids, proteins, and minerals. Several studies have focused on the component effects on larval FA composition following waste treatment, e.g., the effects of bread, seafood, vegetables, and coffee grounds. [27,28,29]; however, the macronutrient effects on larval FA accumulation are largely ignored. Cohn et al. [30] reported that larval lipid contents were extremely increased by glucose and wheat starch components, which highlights the importance of carbohydrates on larval FA transformation. Hoc et al. [21] conducted a deuterated water labeling assay and found that dietary carbohydrates were essential sources of acetyl-CoA, which drove the larval FA biosynthesis; BSFL was highly efficient in the biosynthesis of C12:0 FA, could synthesize ≥C14 SFAs and MUFAs, and could not synthesize PUFAs. Therefore, carbohydrates in food waste may play an important role in the transformation of larval FAs; however, the relationship between waste carbohydrates and larval FAs has not been revealed. Moreover, the oil component was found harmful or less digestible to BSFL [31], whereas BSFL can incorporate PUFAs from diet oils [32,33,34]. The conversion ratio of waste to larval FAs, as well as the optimal time window for larvae harvest engaging both waste FA removal and larval FA accumulation, requires deep investigation. 

Altogether, this study aimed to understand the principal factors that drove the composition and accumulation of larval FAs following food waste treatment and provide an assessment for a feasible time window of the larvae harvest period. Therefore, the FA profiles of larvae and frass, as well as the carbohydrate properties of frass, were examined in a chronological manner, engaging with both original and adjusted food waste substrates.

## 2. Results and Discussion

### 2.1. Larvae Development

**Figure 1:**
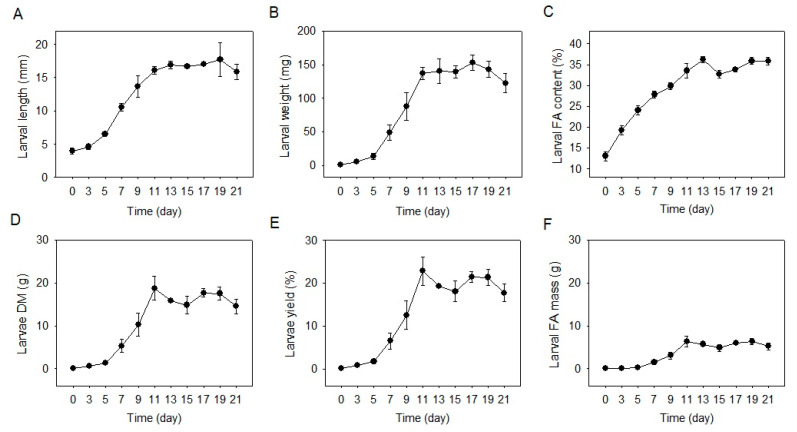
The growth parameters of larvae during the food waste treatment. (**A**), body length; (**B**), body weight: (**C**), body FA content; (**D**), total dry matter of larvae; (**E**), larvae yield; (**F**), total FA mass of larvae. Data are shown as mean ± standard deviation (*n* = 3). FA, fatty acids; DM, dry matter.

The larval body length and weight rapidly increased from 4.0 to 16.1 mm and 0.6–136.7 mg from Day 0–11, maintained at 16.1–17.7 mm and 136.7–152.7 mg from Day 11–19, and decreased to 15.9 mm and 122.3 mg on Day 21, respectively (Figure 1A,B). The larval FA content ascended from 13.0% to 36.2% from Day 0–13 and was kept at 32.7% to 35.9% from Day 13–21 (Figure 1C). The larval total dry matter exhibited the same trend as the body weight, which grew from 0.08 g to 18.8 g from Day 0–11, varied from 14.9 g to 18.8 g from Day 11–19, and declined from 17.6 g to 14.6 g from Day 19–21 (Figure 1D). The larvae yield peaked at 22.8% on Day 11, altered from 18.1% to 22.8% from Day 11–19 and slightly reduced to 17.7% on Day 21 (Figure 1E). The larval FA mass exhibited the same trend as the larval body weight and the total dry matter, which peaked at 6.34 g on Day 11 and maintained at 4.87–6.34 g from Day 11–21, with a slight decrease from 6.32 g to 5.23 g from Day 19–21 (Figure 1F). Upon Day 21, all the larvae showed light brown to dark brown color, which suggested all the larvae had entered the early prepupae stage.

The current larvae fed on food waste exhibited a similar trend in body development and FA accumulation compared with those fed on chicken feed [13,14], except that their development time was relatively longer. Based on the animal feed substrate, Liu et al., Zhu et al., and Li et al. [13,14,15] have all found that the four- or six-day-old larvae rapidly developed and peaked their body weight and fat content in five to eight days; the body weight started to decrease in the changing of early- to late-prepupae stage, while the body fat content began to reduce in the altering of late-prepupae to the early-pupae stage. These similarities imply that BSFL development follows the same growth curve in either food waste or chicken feed substrate. That is, a rapid body weight gain in the early- to middle-larvae stage, a plateau time in the middle-larvae to early-prepupae stage, and a body weight loss in the transfer from early- to late-prepupae stage, associated with a body fat drop in the conversion from late-prepupae to the early-pupae stage. The longer development time of the current larvae than the previous studies [13,14,15] could be attributed to the differences of experimental temperature and complexity of the food waste. Opare et al. [35] reported that high temperatures of 27–30 °C resulted in shorter larval development time than a low temperature of 23 °C. The current experiment was conducted at the ambient temperature of 22–26 °C, which is lower than the reference temperature of 26–27 °C [13,14,15], thus contributing to the longer development time. In the treatment of organic wastes from stores and kitchens, Lalander et al. and Galassi et al. [36,37] found that larvae took 10–14 days and 15–16 days, respectively, to peak the body weight, which are consistent with the current findings of 11 days for larval maximum body weight.

In terms of body FA content, the current BSFL reached 32.7–35.9% FA contents from Day 11–19, which was comparable to the 34.5% FAs (food waste substrate) reported by Ewald et al. [29], slightly higher than the 26.2–30.5% FAs (coffee grounds and dough substrate) found by Fischer et al. [38], and higher than the 24.4–26.1% crude fat (chicken feed substrate) reported by Zhu et al. [14]. The larval FA content reflects the substrate energy and nutrient level [36]. Our findings suggest that the food waste used in this study comprised comparable or relatively higher energy content for BSFL than those studies using organic waste or animal feed substrate.

### 2.2. Waste Reduction and Frass Properties

**Figure 2:**
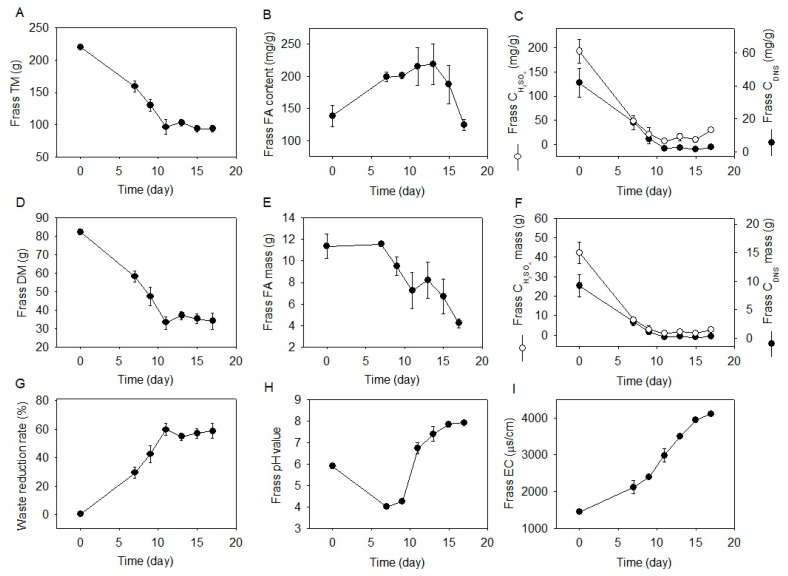
Changes in frass physicochemical properties. (**A**), frass total matter; (**B**), frass FA content; (**C**), frass content of reducing carbohydrate and total carbohydrate; (**D**), frass dry matter; (**E**), frass FA mass; (**F**), frass mass of reducing carbohydrate and total carbohydrate; (**G**), waste reduction rate; (**H**), frass pH value; (**I**), frass EC value. Data are shown as mean ± standard deviation (*n* = 3). TM, total mass; DM, dry matter; FA, fatty acids; EC, electrical conductivity; C_DNS_, reducing carbohydrates; C_H2SO4_, total carbohydrates.

The frass total matter and dry matter rapidly decreased from Day 0–11 and maintained at 93.7–103.4 g and 33.2–37.2 g from Day 11–17, respectively (Figure 2A,D). The waste reduction rate reached 60.0% on Day 11 and varied between 54.9–60.0% from Day 11–17 (Figure 2G).

The frass FA content increased from 138.3 to 219.2 mg/g from Day 0–13 and decreased from 219.2 to 124.5 mg/g from Day 13–17, corresponding to a 10.0% reduction overall (Figure 2B). The frass total FA mass was consistent being at 11.4–11.6 g from Day 0–7, and descended to 4.2 g on Day 17, corresponding to a 63.6% reduction (Figure 2E). The reducing and total carbohydrate content in frass dropped from 41.7 to 2.1 mg/g and from 192.1 to 6.7 mg/g from Day 0–11, respectively, corresponding to a 95.0–96.5% reduction (Figure 2C). The reducing and total carbohydrate mass in frass declined from 9.2 to 0.2 g and from 42.3 to 0.6 g from Day 0–11, respectively, representing a 97.8–98.5% decrease (Figure 2F). The frass pH value decreased from 5.91 to 4.00 from Day 0–7 and increased to 7.92 on Day 17 (Figure 2H). The frass EC value increased continuously from 1440.0 to 4110.0 μs/cm from Day 0–17 (Figure 2I).

Most previous studies focused on waste reduction rates following BSFL treatment, whereas the rear research investigated the degradation process of waste macronutrients. Singh et al. [39], Diener et al. [40], and Lu et al. [41] found that the reduction rates of kitchen, fruit, vegetable, and canteen food waste ranged between 61.96–71.94%, 66.4–78.9%, and 61.0–84.8%, respectively. This study found a comparable waste decomposition rate of 54.9–60.0%, which remained stable from Day 11–17, suggesting that the majority of waste mass was degraded by BSFL in the first 11 days of treatment. Further, this study revealed that waste macronutrients were decomposed in different and various trends compared with the total waste mass, that carbohydrate mass was reduced 97.8–98.5% from Day 0–11, while the FA mass was reduced 63.6% from Day 7–17 (Figure 2E,F), suggesting that waste carbohydrates were degraded faster than waste FAs. These findings indicate that BSFL preferentially consumed waste carbohydrates other than waste oil for larval development, and when the carbohydrates were close to depletion, the BSFL started to use waste FAs. Although the waste reduction rate remained stable from Day 11–19, the optimal time window for larvae harvest should be from Day 17–19 since there was continuous waste FA degradation from Day 7–17 and larval dry mass started to decrease from Day 19–21 (Figure 1). BSFL was not efficient at utilizing FAs, which is in agreement with the findings of Klammsteiner et al. [31]. Further, the pH and EC values changed according to the reduction trend of macronutrients. The pH curve reflected the changes in carbohydrate contents, showing that the frass was acidic during carbohydrate consumption (Day 0–11) and altered to alkaline when the carbohydrates were depleted (Day 11–17). The EC value gradually increased in tandem with continuous macronutrient decomposition and mineral accumulation in frass. 

### 2.3. Fatty Acid Composition and Transformation

**Figure 3:**
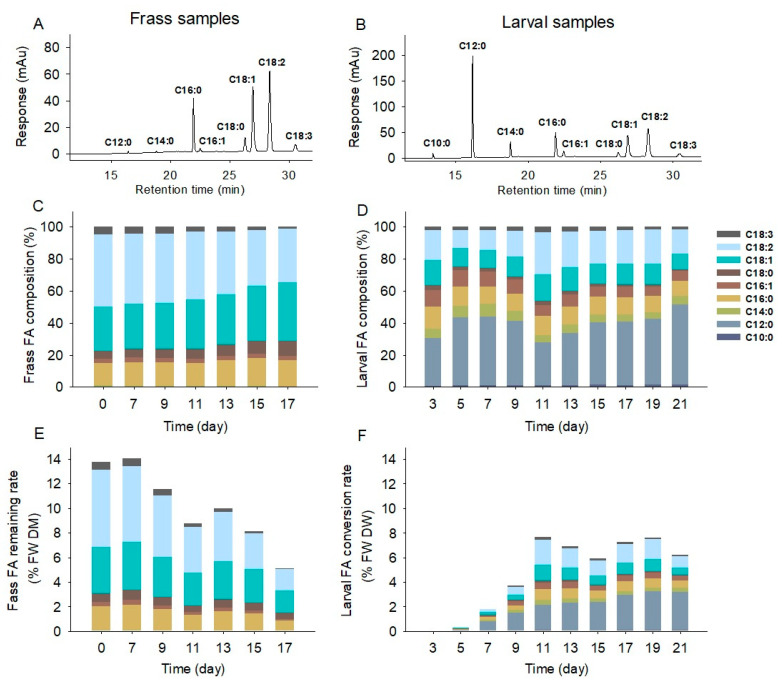
Gas chromatography profiles (**A**,**B**), FA composition (**C**,**D**), and FA conversion rates (**E**,**F**) of frass and larvae in the food waste treatment. (**C**–**F**), data are the means of triplicates; the legend in D is for all subfigures (**C**–**F**). FW, food waste; DM, dry matter; % FW DM, percentage of food waste dry matter.

The FAs of food waste and frass were mainly composed of linoleic acid (C18:2), oleic acid (C18:1), and palmitic acid (C16:0) (Figure 3A), while larval FAs were dominated by lauric acid (C12:0) and followed by linoleic acid (C18:2), oleic acid (C18:1), and palmitic acid (C16:0) (Figure 3B). In the frass FA profiles, the C18:1 FA increased from 27.5% to 36.2%, the C18:2 FA decreased from 45.1% to 33.4%, and the C16:0 FA ranged between 13.9% and 17.6% from Day 0–17 (Figure 3C). The larval FA profiles exhibited two patterns of FA changes during Day 0–11 and Day 11–21 (Figure 3D). Throughout Days 0–11, the C12:0 ratio increased first and then decreased, varying between 26.8–43.1%, while the C18:2 ratio decreased first and thereafter increased, ranging between 11.3–26.1% (Figure 3D); from Day 11–21, the C12:0 FA gradually ascended from 26.8% to 49.8%, while the C18:2 FA continuously descended from 26.1% to 15.2% (Figure 3D). The FA mass changes in both frass and larvae were further illustrated in Figure 3E,F. Overall, the FA mass in the initial food waste accounted for 13.8% of food waste dry matter (% FW DM). As the treatment went on, the frass FA mass remained stable from Day 0–7 and started to decrease from 14.0% FW DM to 5.1% FW DM from Day 7–17, corresponding to a 63.6% reduction rate (Figure 3E). The larval FA mass increased from 0 to 7.7% FW DM from Day 0–11 and maintained at 5.9% FW DM–7.7% FW DM from Day 11–21 (Figure 3F).

The current study exhibited different dynamic patterns of larval FA composition in BSFL feeding on food waste compared with those feeding on chicken feed [13,14], although their growth patterns in body weight development were similar. Liu et al. and Zhu et al. [13,14] reported that the larvae feeding on chicken feed showed a continuous increase in the C12:0 ratio (16.4% to 73.4%) and a decrease in the C18:2, C18:1, and C16:0 ratios (31.4% to 3.4%) in the development of six-day-old larvae to late-prepupae. However, the current larvae feeding on food waste exhibited that the C12:0 ratio in the larval FA pool was not monodirectional, whereas it fluctuated over the development time. These differences could be highly attributed to the larval metabolism character and substrate component differences. Both Hoc et al. [21] and Cohn et al. [30] reported that BSFL were efficient in the transformation of starch to larval FAs, which incorporated both the catabolism process of glucose to acetyl-CoA and the anabolism process of acetyl-CoA to C12:0 FA. The chicken feed was typically composed of 60% corn meal, 25% soybean meal, and 5% oil [16], while the current food waste comprised 19.2% total carbohydrates and 13.8% of FA mass (Figure 2). These differences suggest that the chicken feed had a higher starch ratio and lower FA ratio than the current food waste, which explained the continuous increase in the C12:0 ratio in the larvae fed on chicken feed since the starch was the major source for larval FA biosynthesis. On the contrary, the current larval FAs were synthesized from both the waste carbohydrates and FAs, which resulted in the fluctuation in the C12:0 ratio. As the BSFL could produce C12:0 from carbohydrates and could also transform C12:0 from long-chain FAs [21], the variation of carbohydrates and FAs in the frass explained the dynamics of the C12:0 ratio in the larval FA pool. From Day 0–7, the waste carbohydrates were sufficient and consumed quickly, corresponding to the increase in larval C12:0 ratio from 29.9% to 43.1%. From Day 7–11, the waste carbohydrates rapidly decreased and were close to depletion, associated with the decrease in the C12:0 ratio from 43.1% to 26.8%. From Day 11–17, the waste carbohydrates were depleted, and the larvae began to use waste FAs as nutrients. Long chain (≥C16) FAs were decomposed to C12:0, which led to an increase in the C12:0 ratio from 26.8% to 49.8%. Therefore, it is highly likely that the larval C12:0 was primarily biosynthesized from the waste carbohydrates and further from the waste FAs. Bearing in mind that the high UFA proportions are conducive to the larval FA feed application, the current BSFL had higher UFA ratios (C18:2 11.3–26.1%, C18:1 11.3–16.8%) than those of chicken feed larvae, which could be highly attributed to the waste FA content. The BSFL could partially synthesize MUFAs and only accumulate PUFAs from the substrate [21,31,32,33]; the high content of C18:2 and C18:1 FA in the food waste over the treatment period contributed to the UFA proportions in the larvae.

The shift of nutrient substrate from carbohydrates to FAs resulted in changes in larval FA profiles. This phenomenon is similar to the findings observed in the lactose operon of *Escherichia coli*. The shift of nutrients from glucose to lactose resulted in a fluctuation of the cell growth curve and the transcriptional regulation of lactose metabolism genes in *Escherichia coli* [42]. The alternation of nutrient substrates for BSFL could also initiate gene regulation of larval transcriptome, especially the lipid metabolism genes. The larval transcriptome during the nutrient shift period should be further investigated to reveal the genetic mechanism underlying lipid metabolism dynamics.

### 2.4. Correlations of Larval Fatty Acid and Frass Macronutrient

**Figure 4:**
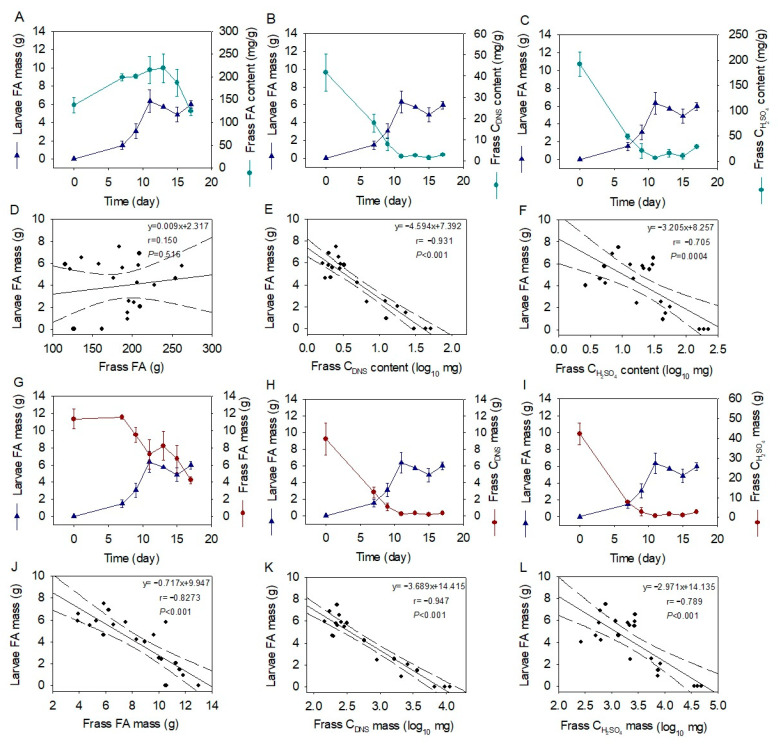
Chronological changes in larval FA mass and frass macronutrient properties (**A**–**C**,**G**–**I**) as well as their relationships (**D**–**F**,**J**–**L**). Data are shown as mean ± standard deviation (*n* = 3). Solid lines are the fitted curves, and dashed lines are the ranges of 95% confidence intervals. FA, fatty acids; C_DNS_, reducing carbohydrates; C_H2SO_4__, total carbohydrates.

The changes in larvae FA mass were plotted associated with the changes in frass FA content, FA mass, reducing carbohydrate content, reducing carbohydrate mass, carbohydrate content, and carbohydrate mass, as shown in Figure 4A–C,G–I. Their correlations are presented in Figure 4D–F,J–L. The larvae FA mass was not correlated with the frass FA content (r = 0.150, *p* = 0.516, Figure 4D), whereas it was linearly correlated with the frass FA mass (r = −0.827, *p* < 0.001, Figure 4J). Further, the larvae FA mass was linearly correlated with the log value of frass-reducing carbohydrate content (r = −0.931, *p* < 0.001, Figure 4E) and its mass (r = −0.947, *p* < 0.001, Figure 4K). Additionally, the larvae FA mass was linearly correlated with the log value of frass carbohydrate content (r = −0.705, *p* < 0.001, Figure 4F) and its mass (r = −0.789, *p* < 0.001, Figure 4L). 

The frass FA mass, reducing carbohydrate mass, and carbohydrate mass exhibited significant correlations with the larval FA mass. However, the r values showed that the relationships ranked as reducing carbohydrate mass > FA mass > carbohydrate mass. This result is reasonable since the BSFL primarily consumes starch for FA constitution [21,30] and starts to utilize FAs when the reducing carbohydrates are depleted. The frass carbohydrate mass was less correlated to the larval FA mass than the reducing carbohydrate mass since not all the carbohydrates, such as cellulose and hemicellulose, could be efficiently consumed by the BSFL. The log value of the reducing carbohydrate mass exhibited a better relationship with the larval FA mass than the original value, indicating that the reducing carbohydrates were consumed fast in an exponential manner to support the larval growth, which was in agreement with the rapid accumulation of larval C12:0 mass as well as the total FA mass. Therefore, both the content and the mass of reducing carbohydrates are good indicators for the waste decomposition process, which could be explored in other practical monitoring of food waste treatment. As in the case of the current study, the frass-reducing carbohydrates were further determined in the study of substrate adjustment assay. 

### 2.5. Fatty Acid Transformation of Adjusted Substrate 

**Figure 5:**
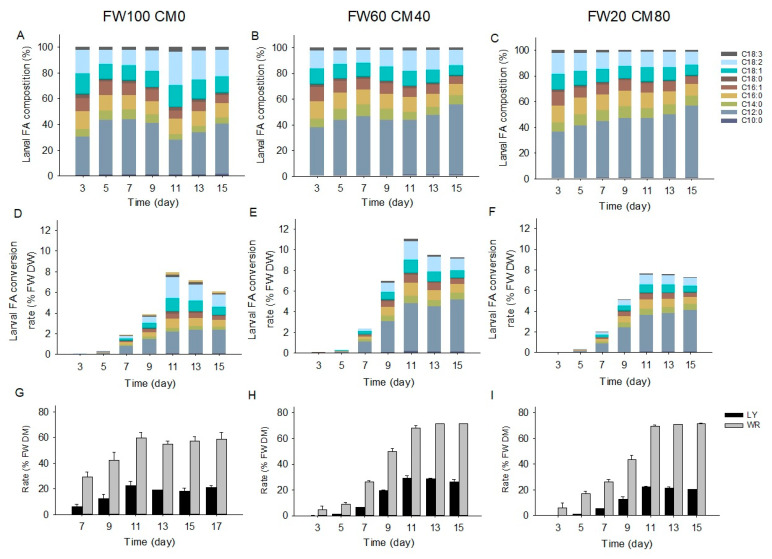
Larval FA composition (**A**–**C**), larval FA conversion rate (**D**–**F**), larvae yield, and waste reduction rate (**G**–**I**). (**A**–**F**), data are means of triplicates; the legend in C is for subfigures (**A**–**F**). (**G**–**I**), data are shown as mean ± standard deviation (*n* = 3); the legend in I is for subfigures (**G**–**I**). FW100 CM0, 100% food waste; FW60 CM40, 60% food waste and 40% corn meal; FW20 CM80, 20% food waste and 80% corn meal. FA, fatty acids; FW, food waste; CM, corn meal; % FW DM, percentage of food waste dry matter; LY, larvae yield; WR, waste reduction rate.

The adjustment of food waste substrate resulted in deviations in larval FA chronological pattern, as well as changes in larval FA conversion rates and waste reduction rates (Figure 5). In terms of larval FA patterns, fluctuations in C12:0 ratios were observed in both the FW 100 CM0 and FW60 CM40 groups, whereas they were not found in the FW20 CM80 group (Figure 5A–C). These results suggest that there were still nutrient shifts for BSFL from carbohydrates to FAs in the FW 100 CM0 and FW60 CM40 groups but not in the FW20 CM80 group. The FW20 CM80 larvae were mainly fed on corn starch; thus, the FA chronological pattern was comparable to the chicken feed assay [13,14], and the C12:0 proportion continuously increased over the treatment. In terms of larval FA conversion rates, the FW100 CM0 larvae accumulated 5.9–7.7% FW DM of FAs from Day 11–15 (Figure 5D), while the FW60 CM40 larvae achieved 9.3–11.1% FW DM of FAs (Figure 5E), and the FW20 CM80 larvae gained 7.3–7.6% FW DM of FAs (Figure 5F). These results indicate that the FW60 CM40 group was superior to the FW100 CM0 group for the larvae FA conversion and was better than the FW20 CM80 group as well. Although carbohydrates are essential substrates for FA biosynthesis, excess carbohydrate supplement, such as in the FW20 CM80 group, may result in decreased FA conversion due to unbalanced nutrition for the larvae. In terms of waste reduction performance and larvae yield, the FW60 CM40 group showed a 68.3–71.4% waste reduction rate (11–15 days), while the FW20 CM80 group exhibited a 69.7–71.4% waste reduction rate (11–15 days), which were all higher than the 54.9–58.7% reduction rate of the FW100 CM0 group (11–15 days). These results could likely be due to the replacement of food waste with corn meal, which increased the starch and decreased the cellulose in the FW60 CM40 and FW20 CM80 substrates. The larvae yield of the FW60 CM40 group reached 26.8–29.4% (11–15 days), which was higher than the 20.3–22.3% of the FW20 CM80 group (11–15 days) and the 18.1–21.4% of the FW100 CM0 group (11–15 days). These results indicate that the BSFL in the FW60 CM40 group had better nutritional condition than the FW20 CM80 and FW100 CM0 larvae, and optimization of substrate composition could increase larvae yields as well as the FA conversion performance.

**Figure 6:**
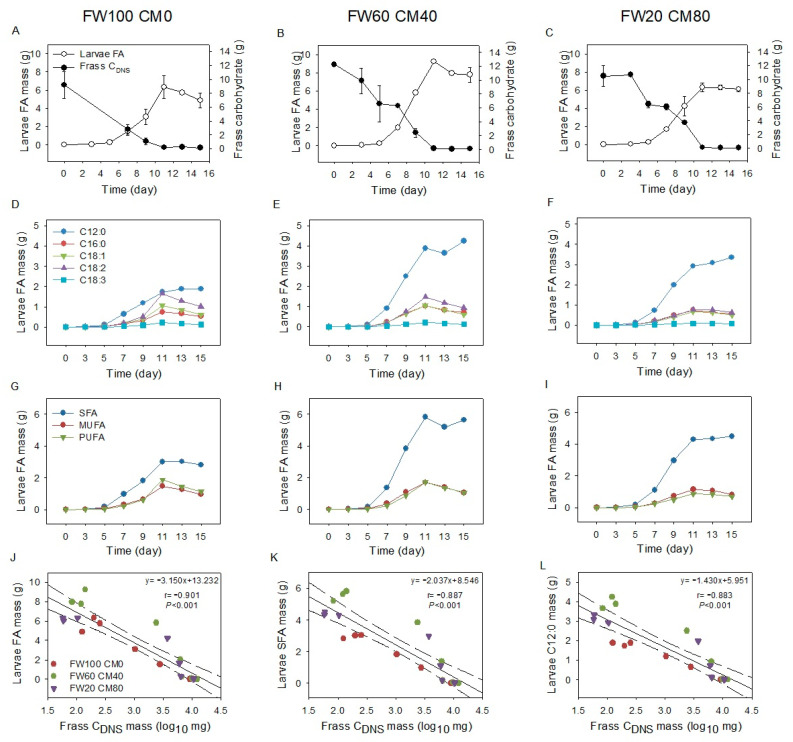
Chronological changes of larval FA mass and frass reducing carbohydrate mass (**A**–**C**), changes of larval FA mass in different FA species (**D**–**I**), and relationships of larval FA mass and frass reducing carbohydrate mass (**J**–**L**). (**A**–**C**), data are shown as mean ± standard deviation (*n* = 3) with the same legend in (**A**). (**D**–**F**), data are the mean of triplicates with the same legend in (**D**). (**G**–**I**), data are the mean of triplicates with the same legend in 6G. J–L, data are the mean of triplicates with the same legend in (**J**); solid lines are the fitted curves, and dashed lines are the ranges of 95% confidence intervals. FW100 CM0, 100% food waste; FW60 CM40, 60% food waste and 40% corn meal; FW20 CM80, 20% food waste and 80% corn meal; FA, fatty acids; C_DNS_, reducing carbohydrates; SFA, saturated fatty acid; MUFA, mono-unsaturated fatty acid; PUFA, polyunsaturated fatty acid.

**Figure 7:**
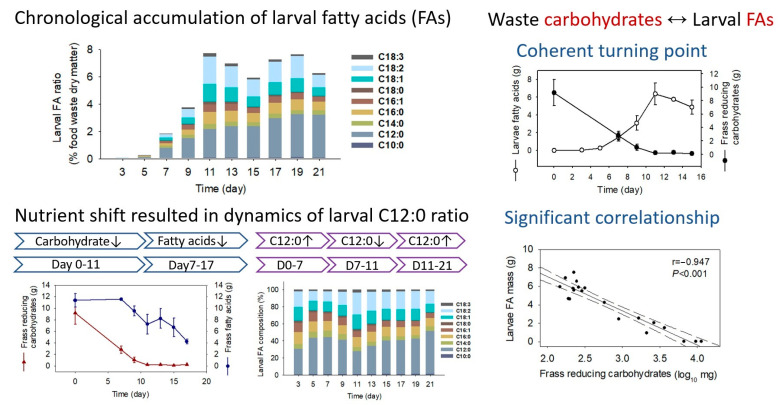
Summary of the chronological and carbohydrate-dependent transformation of FAs in the larvae of black soldier fly following food waste treatment.

Chronological changes in larval FA mass and frass-reducing carbohydrate mass were plotted in Figure 6A–C. The decrease in frass carbohydrate mass simultaneously occurred with the increase in larval FA mass, and the turning points for both curves overlapped on Day 11 regardless of the substrate difference (Figure 6A–C). As food waste was replaced with the corn meal, the larval C12:0 mass significantly increased, and the larval C18:1 and C18:2 mass significantly decreased (Figure 6D–E), associated with the ascended SFA proportions and descended UFA proportions (Figure 6G–I) in the comparison of FW60 CM40, FW20 CM80 larvae and those of FW100 CM0 larvae. These results confirm the findings that the larval C12:0 FA and SFAs were mainly derived from starch components in the waste [21,30], and the larval UFAs were largely accumulated from the waste FAs [31,32,33]. The correlation analysis further proved the significant relationships between the frass-reducing carbohydrate mass (log value) associated with the larval FA mass (r = −0.901, *p* < 0.001), the larval SFA mass (r = −0.887, *p* < 0.001), and the larval C12:0 mass (r = −0.883, *p* < 0.001) across varied substrates (Figure 6J–L). Thus, BSFL preferentially uses reducing carbohydrates for C12:0 synthesis in vivo. The substrate carbohydrate mass substantially affects larval FA composition and transformation, associated with the reducing carbohydrates positively correlated with the larval C12:0 ratio (Figure 7). The substrate FA mass drives the proportion and accumulation of UFAs in BSFL. The coherent turning points of larval FA and frass-reducing carbohydrate curves suggest that reducing carbohydrates is a good indicator for monitoring the decomposing process of food waste. 

## 3. Materials and Methods

### 3.1. Materials and Reagents

BSF eggs were purchased from Langhao Environmental Technology Co., Ltd. (Nanjing, China). Food waste was collected from the university canteen, which comprised cooked foods with components of rice, noodles, tofu, eggs, vegetables, meats, fish, etc. Wheat bran, soybean meal, and corn meal were purchased from a local store. Analytical or chromatographical grade chemical reagents were provided by Maclin Biochemical Technology Co., Ltd. (Shanghai, China), which included: petroleum ether, n-hexane, methanol, H_2_SO_4_, phenol, glucose, soluble starch, decanoic acid methyl ester (C10:0), lauric acid methyl ester (C12:0), myristic acid methyl ester (C14:0), palmitic acid methyl ester (C16:0), palmitoleic acid methyl ester (C16:1), stearic acid methyl ester (C18:0), oleic acid methyl ester (C18:1), linoleic acid methyl ester (C18:2), linolenic acid methyl ester (C18:2), and linolenic acid methyl ester (C18:3). DNS (3,5-dinitrosalicylic acid) reagent was purchased from Yixun Biotechnology Co., Ltd. (Nanjing, China). 

### 3.2. Food Waste Treatment

The experiment was carried out in the laboratory of Dalian University of Technology (40°41′20.26″ N, 122°7′15.17″ E) in September–October 2021 at 22–26 °C ambient temperatures and approximately 12 daytime hours. The BSF eggs were hatched in a substrate containing soybean meal, corn meal, and wheat bran in a 6:3:1 ratio with 70% moisture content for 6–8 d. The emerging larvae were sieved out and weighed 0.0591 g per 100 individuals. The food waste was fully mixed by a kitchen blender and split into transparent plastic boxes. Each box was filled with 200 g of food waste, 20 g of wheat brain, and 480 larval individuals (0.2837 g). The boxes were 1250 mL in volume with several holds on the lids for passive aeration. A total of 30 parallel boxes were prepared. On Days 3, 5, 7, 9, 11, 13, 15, 17, 19, and 21, triplicate boxes were collected, and the larvae and frass were separated manually, weighted, and stored at −20 °C. Larval samples of all time points were subjected to the detection of body parameters and fatty acid properties. Frass samples of Days 7, 9, 11, 13, 15, and 17 were used for the determination of physiochemical properties. 

Food waste components were further adjusted for analysis of carbohydrate effects on the larval bioconversion process. Three substrates were set as 100% food waste (FW100 CM0), 60% food waste and 40% corn meal (FW60 CM40), and 20% food waste and 80% corn meal (FW20 CM80). Percentages of each component were based on their wet weight. The FW100 CM0 group was the food waste treatment carried out above. The FW60 CM40 and FW20 CM80 groups were performed in the same manner as the experiments above, except that the waste components and sampling time points were adjusted. The food waste was still the university canteen waste, whereas the corn meal was prepared by mixing corn meal flour and water in a 3:7 ratio and cooking in a rice cooker for 0.5 h. When the substrates were mixed thoroughly, 21 parallel boxes were prepared for the FW60 CM40 and FW20 CM80 groups, respectively. The sampling time points were set as Days 3, 5, 7, 9,11, 13, and 15. At each time point, triplicate boxes were collected, and the larvae and frass were manually separated and weighted. The larvae were further analyzed for body FA contents and compositions, and the frass samples were further determined for the properties of reducing carbohydrates.

### 3.3. Fatty Acid Analysis

The samples of food waste, BSFL, and frass were determined for their fatty acid contents and compositions. All samples were firstly dried in a 60 °C oven for 16 h and then extracted for crude fat with petroleum ether as described by Li et al. [43]. The crude fat was derivatized into fatty acid methyl esters (FAMEs) as described by Saadoun et al. [27]. Briefly, 100 mg of crude fat was added with 1 mL methanol containing 5% (*v*/*v*) HCl in a screw tube and kept at 100 °C for 1 h to generate FAMEs. The reaction solution was then added with 500 μL n-hexane and 400 μL deionized waster to collect the organic upper layer. The 500 μL n-hexane was additionally supplied twice with the three sections of upper layers combined and fixed to 5 mL. The derivatized FAMEs were then analyzed for the FAME composition using gas chromatography (GC) equipment and the relevant external standards. The Shimazu GC-2014C equipment (Shimazu Instruments Ltd., Suzhou, China) was equipped with AOC-20i Plus automatic injector and a flame ionization detector (FID). An FFAP capillary column (Zhongke Kaidi Co. Ltd., Lanzhou, China) with parameters of 50 m × 0.25 mm × 0.50 μm (length × inner diameter × film thickness) was employed. Ultrapure nitrogen was used as the carrier gas at a flow rate of 1 mL/min. Samples were injected with 1 μL volume through the port at a 30:1 split ratio with 250 °C injection temperature and 250 °C detector temperature. The GC program was set as follows: initial temperature of 70 °C, ascending from 70 °C to 240 °C at 10 °C/min and holding at 240 °C for 15 min (32 min in total). The FAMEs were identified based on their retention times compared with the reference FAMEs (Section 3.1) under the same GC conditions. The FAMEs were quantified by comparing individual peak areas with the peak areas of external standards using GC Solution software Version 2.3 (Shimadzu, Kyoto, Japan). External standards were prepared in n-hexane solvent with 10-fold series diluted concentrations. The FA amounts were calculated based on the respective FAME matters, and the total FA amounts were obtained by the sum of individual FA matters. 

### 3.4. Physiochemical Property Analysis

Apart from the FA content, the frass samples were examined for moisture content, pH value, electrical conductivity (EC), reducing carbohydrate content, and carbohydrate content. Frozen samples were melted to the ambient temperature and used for the detection. The frass moisture was measured through oven-drying at 105 °C for 2 h [41]. The frass pH and EC value were detected once the sample was dissolved in deionized water at a 1:10 (*w*/*v*) ratio for 30 min. A LE420 pH meter and a 731-ISM EC meter (Mettler-Toledo GmbH, Zurich, Switzerland) were used for the detection [44]. The frass samples were further mixed with deionized water at a 1:10 (*w*/*v*) ratio and kept at ambient temperature for 30 min. The supernatant was collected and used for the detection of soluble carbohydrates. The reducing carbohydrate content was determined using the DNS reagent with glucose as the standard, as described previously [45,46]. The carbohydrate content was examined with the H_2_SO_4_-phenol method using soluble starch as the standard, as detailed in previous studies [45,46]. 

### 3.5. Conversion Efficiency Estimates

Waste conversion efficiency was evaluated using Equations (1) and (2). Larval and frass FA contents were estimated using Equation (3). FA compositions were calculated using Equation (4). The larvae FA conversion rate and frass FA remaining rate were estimated using Equations (5) and (6), respectively.

(1) $$\mathrm{Larvae}\;\mathrm{Yield}\;\left(\mathrm{LY};\%\right)=\frac{{\mathrm{DM}}_{L}}{{\mathrm{DM}}_{W}}\times 100\%\;$$

(2) $$\mathrm{Waste}\;\mathrm{Reduction}\;\mathrm{Rate}\;\left(\mathrm{WR};\;\%\right)=\left(1-\frac{{\mathrm{DM}}_{F}}{{\mathrm{DM}}_{W}}\right)\times 100\%\;$$

(3) $$\mathrm{Fatty}\;\mathrm{Acid}\;\mathrm{Content}\;\left({\mathrm{FA}}_{L}{\;\mathrm{or}\;\mathrm{FA}}_{F};\%\right)=\frac{\sum_{i}C_{i}}{\mathrm{DM}}\times 100\%\;$$

(4) $$\mathrm{Fatty}\;\mathrm{Acid}\;\mathrm{Composition}\;\left({\mathrm{FA}}_{i};\;\%\right)=\frac{C_{i}}{\sum_{i}C_{i}}\times 100\%$$

(5) $$\mathrm{Larvae}\;\mathrm{FA}\;\mathrm{conversion}\;\mathrm{rate}\;\left(\%\;\mathrm{FW}\;\mathrm{DM}\right)=\frac{\sum_{i}({\mathrm{DM}}_{L}\times {\mathrm{FA}}_{i})}{{\mathrm{DM}}_{W}}\times 100\%$$

(6) $$\mathrm{Frass}\;\mathrm{FA}\;\mathrm{remaining}\;\mathrm{rate}\;\left(\%\;\mathrm{FW}\;\mathrm{DM}\right)=\frac{\sum_{i}({\mathrm{DM}}_{F}\times {\mathrm{FA}}_{i})}{{\mathrm{DM}}_{W}}\times 100\%$$

where DM_W_ is the total dry matter of food waste, g; DM_F_ is the total dry matter of frass, g; DM_L_ is the total dry matter of larvae, g; C_i_ is the mass of individual FA in the detected sample, g; DM is the dry matter of sample used for the FA detection, g; FA_i_, is the proportion of individual FA in the detected sample, %; the unit of % FW DM in Equations (5) and (6) represents the percentage of food waste dry matter. 

### 3.6. Statistical Analyses

Statistical analyses and figures were prepared using the SigmaPlot software version 14.0. Group differences were compared using one-way variance analysis (ANOVA). The correlation analysis was conducted using the linear regression wizard. Statistical significance was defined as *p* < 0.05.

## 4. Conclusions

Waste carbohydrates were decomposed by 98% after 11 days of treatment, while the waste FAs were reduced by 64% after 17 days. The BSFL primarily utilized waste carbohydrates for FA bioconversion and transferred them to the waste FAs while the carbohydrates were almost depleted. The shift of nutrient substrate resulted in changes in larval FA profiles, especially the C12:0 proportions. The reducing carbohydrates were consumed fast in an exponential manner to support the larval growth, which was principally correlated with the larval FA accumulation (r = −0.901, *p* < 0.001) and C12:0 in vivo synthesis (*r* = −0.883, *p* < 0.001). The waste carbohydrates accounted for larval FA composition and transformation, while the waste FAs accounted for larval UFA proportions. The reducing carbohydrate is a good indicator for monitoring the decomposing process of food waste. To incorporate both the high yield of larvae FAs and deep degradation of waste FAs, BSFL should be harvested at the Day 17–19 stage. The current findings provide larval FA profiles in a chronological manner, which is expected to guide the industrial application of BSFL technology for food waste treatment. 

## Data Availability

Data are available through email requirements.

## Sample Availability

Samples of the larval and frass mass are available from the authors.
